# Autoimmune Lymphoproliferative Syndrome in Children with Nonmalignant Organomegaly, Chronic Immune Cytopenia, and Newly Diagnosed Lymphoma

**DOI:** 10.4274/tjh.galenos.2020.2020.0618

**Published:** 2021-06-01

**Authors:** Zühre Kaya, Melek Işık, Nihan Oruklu, Serap Kirkiz, Emin Ümit Bağrıaçık, Luis M. Allende, María J. Díaz-Madroñero, Raquel Ruiz-García, Faruk Güçlü Pınarlı, Pınar Göçün Uyar, Ülker Koçak

**Affiliations:** 1 Gazi University Faculty of Medicine, Department of Pediatric Hematology, Ankara, Turkey; 2 Gazi University Faculty of Medicine, Department of Immunology and Life Science Research Center, Ankara, Turkey; 3 Immunology Department and Research Institute i+12, Hospital Universitario 12 de Octubre, Madrid, Spain; 4 Gazi University Faculty of Medicine, Department of Pediatric Oncology, Ankara, Turkey; 5 Gazi University Faculty of Medicine, Department of Pathology, Ankara, Turkey

**Keywords:** Autoimmune lymphoproliferative syndrome, Immune cytopenia, Lymphoma

## Abstract

This study investigated the frequency of and predictive factors for autoimmune lymphoproliferative syndrome (ALPS) in children with lymphoma, chronic immune cytopenia, and nonmalignant organomegaly. Thirty-four children with suspected ALPS (n=13, lymphoma; n=12, immune cytopenia; n=9, nonmalignant organomegaly) were included. Double-negative T-cells, lymphocyte apoptosis, and genetic findings were analyzed. Patients were stratified into two groups as proven/probable ALPS and clinically suspected patients according to the ALPS diagnostic criteria. Of the 34 patients, 18 (53%) were diagnosed with proven/probable ALPS. One patient had a mutation (c.652-2A>C) in the *FAS* gene. The remaining 16 (47%) patients were defined as clinically suspected patients. Predictive factors for ALPS were anemia and thrombocytopenia in patients with lymphoma, splenomegaly and lymphadenopathy in patients with immune cytopenia, and young age in patients with nonmalignant organomegaly. ALPS may not be rare in certain risk groups. Our study indicates that screening for ALPS may be useful in children having lymphoma with cytopenia at diagnosis, in those having nonmalignant organomegaly with immune cytopenia, and in those having chronic immune thrombocytopenic purpura or autoimmune hemolytic anemia with organomegaly developing during follow-up.

## Abstract (tr)

Bu çalışmanın amacı malign olmayan organomegali, kronik immün sitopeni ve lenfomalı çocuklarda otoimmün lenfoproliferatif sendrom (OILS) sıklığını ve belirleyici faktörlerini araştırmaktır. Bu çalışmaya OILS şüpheli 34 hasta dahil edildi (13 hasta lenfoma, 5 hasta otoimmün hemolitik anemi, 7 hasta kronik immün trombositopenik purpura ve 9 hasta malign olmayan organomegali). Çift negatif T-hücreler, lenfosit apoptozis ve genetik bulgular analiz edildi. Hastalar OILS tanı kriterlerine göre kesin ve yüksek olasılıklı OILS’li hastalar ve klinik şüpheli OILS’li hastalar olarak iki gruba ayrıldı. Çalışmaya dahil edilen 34 hastanın, 18’i (%53) kesin ve yüksek olasılıklı OILS’di. Malign olmayan organomegalisi olan bir çocukta *FAS* geninde mutasyon (c.652-2A>C) saptandı. Klinik şüpheli hasta 16 (%47) idi. OILS için belirleyici faktörler; lenfomalı hastalarda anemi ve trombositopeni, kronik immün sitopenili hastalarda splenomegali ve lenfadenopati; malign olmayan organomegalisi olan hastalarda genç yaş idi. OILS belirli risk gruplarında nadir olmayabilir. Çalışmamız, ilk tanıda sitopenisi olan lenfomalı çocuklarda, immün sitopenisi olan nonmalign organomegalili çocuklarda ve izlem sırasında organomegalisi gelişen kronik immün trombositopenik purpura ve otoimmün hemolitik anemili çocuklarda OILS taramasının yararlı olabileceğine işaret etmektedir.

## Introduction

Autoimmune lymphoproliferative syndrome (ALPS) is characterized by nonmalignant organomegaly, immune cytopenia, and an increased risk for lymphoma, as well as mutation in the *FAS*-mediated apoptotic pathway [1,2,3,4,5,6,7,8,9,10]. Few studies have considered the identification of ALPS in certain populations, such as patients with Evans syndrome or lymphoma [11,12,13,14,15,16].

The aim of the present study was to investigate the frequency and predictive factors of ALPS in children with recently diagnosed lymphoma, chronic nonmalignant organomegaly, and chronic immune cytopenia.

## Materials and Methods

**Figure 1:**
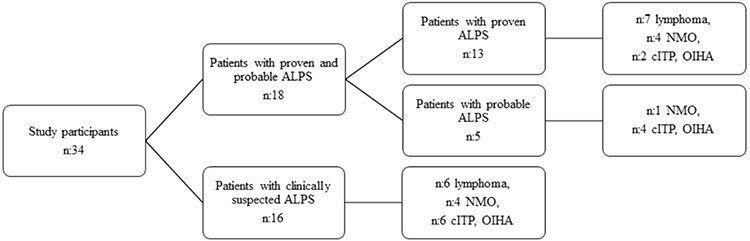
Flow chart of the study participants. ALPS: Autoimmune lymphoproliferative syndrome; NMO: nonmalignant organomegaly; cITP: chronic immune thrombocytopenic purpura; OIHA: autoimmune hemolytic anemia.

In total, 34 consecutive patients were included in this study with a two-stage cross-sectional design: those with nonmalignant organomegaly, chronic immune thrombocytopenic purpura (cITP), or autoimmune hemolytic anemia (AIHA) (n=21) between March 2011 and April 2013, and those with newly diagnosed lymphoma (n=13) between June 2013 and March 2015. Patients were also stratified into two groups as proven/probable ALPS (Group 1, n=18) and clinically suspected patients (Group 2, n=16) according to the ALPS diagnostic criteria [17] (Figure 1). The institutional review board approved the study.

Serum vitamin B12 (>1500 ng/L) and immunoglobulin levels, soluble *FAS* ligand (>200 pg/mL), and interleukin (IL)-10 levels (>20 pg/mL) were measured. Double-negative T-lymphocytes (DNTs; CD3+ T-cell receptor (TCR) αβ+ CD4-, and CD8- DNTs ≥2.5% of the patient’s CD3+ lymphocyte count) were analyzed by flow cytometry [17]. Apoptotic cells were detected by flow cytometry using annexin V-FITC [18]. Nine exons of the *FAS* gene were analyzed by Sanger sequencing. Data analysis was performed using SPSS 15.0.

## Results

The demographic data for ALPS are summarized in Table 1. Of the 34 patients enrolled, 18 (53%) fulfilled the diagnostic criteria for proven ALPS (n=13; 38%) or probable ALPS (n=5; 15%) in Group 1. The remaining 16 (47%) were clinically suspected patients in Group 2. There were significant differences in terms of age between Group 1 and Group 2 (p<0.05). The median age of the patients with nonmalignant organomegaly in Group 1 was significantly lower than that of the nonmalignant organomegaly patients in Group 2 (3 vs. 10 years; p<0.05). The proportions of patients with splenomegaly and lymphadenopathy were significantly higher among the cITP and AIHA subgroups in Group 1 than among the cITP and AIHA subgroups in Group 2 (p<0.05). The proportions of patients with anemia and thrombocytopenia were significantly higher among the lymphoma subgroups in Group 1 than among the lymphoma subgroups in Group 2 (p<0.05).

All relevant data of the 18 patients with proven and probable ALPS are given in Table 2. Of them, 7 (38%) had lymphoma, 5 (28%) had nonmalignant organomegaly, 4 (22%) had cITP, and 2 (12%) had AIHA. Of the seven children with lymphoma, histopathological examination revealed five with Hodgkin lymphoma. Only two of them were positive for Epstein-Barr virus (EBV). Heterozygous splicing mutation in the *FAS* gene (c.652-2A>C in intron 7) was identified in Case 10 as shown in Table 2. The *FAS* mutation rate was found to be 20% among patients with nonmalignant organomegaly (n=5).

Five of the 18 children in Group 1 had been scheduled for splenectomy for massive splenomegaly. Splenectomy was canceled after the diagnosis of ALPS. Three of them responded to steroids and mycophenolate mofetil (MMF), one was unresponsive to steroids and MMF but responded to sirolimus, and one received an allogeneic stem cell transplantation. The remaining seven patients with lymphoma received chemotherapy. Four patients with cITP received mostly on-demand treatment with either steroids or IVIG. Two patients with AIHA received steroids and rituximab, which initially controlled the anemia. MMF was given to both patients who were diagnosed with cITP and AIHA (Cases 13 and 17 in Table 2).

### Predictive Factors for ALPS

Presence of anemia (odds ratio [OR]: 3.2; 95% confidence interval [CI]: 1.0-11.4) and thrombocytopenia (OR: 4.2; 95% CI: 1.4-27.2) in patients with newly diagnosed lymphoma, presence of splenomegaly (OR: 4.1; 95% CI: 1.2-13.2) and lymphadenopathy (OR: 7.0; 95% CI: 1.1-42.1) in patients with chronic immune cytopenia, and young age (OR: 2.0; 95% CI: 3.4-12.9) in patients with nonmalignant organomegaly were identified as predictive factors for ALPS.

## Discussion

Patients with ALPS have heterogeneous phenotypes that can mimic malignancy and infectious or autoimmune diseases. Long-term follow-up studies demonstrated ALPS mutation in 15% and 85% of involved subjects [3,7,8,9,10]. In this study, proven or probable ALPS was recorded in 53% of suspected patients. However, the *FAS* mutation rate was found to be 20% among patients with nonmalignant organomegaly.

Lymphadenopathy and splenomegaly are the most common clinical signs of ALPS, as described in our study [19]. Most patients with type Ia develop lymphoproliferation at a median age of 1.8 years [20]. The same clinical pattern was also described incidentally in a 1-year-old girl with *FAS* mutation in our study. However, the median age at presentation was 4.9 years in patients with undefined ALPS type III [20]. Accordingly, we found the median diagnostic age as 3 years in undefined ALPS patients with nonmalignant organomegaly. Our findings indicate that patients with lymphoproliferation detected incidentally in the infancy period should be closely monitored for ALPS.

Autoimmunity is the second most common sign with the highest probability of requiring medical intervention. The frequencies of ALPS in the subjects with chronic immune cytopenia and in patients with Evans syndrome were 37% and 45%, respectively [7,8,9,10,11,12,13,19]. Similarly, we found ALPS in 34% of patients in Group 1. Children with immune cytopenia with the occurrence of lymphadenopathy/splenomegaly during follow-up were approximately 4- to 7-fold more likely to develop ALPS. These findings indicate that lymphadenopathy/splenomegaly may not appear simultaneously in patients with chronic immune cytopenia. Furthermore, positive Coombs tests and hypergammaglobulinemia are frequently observed in patients with Evans syndrome [11,12,13]. We observed that nearly half of the ALPS patients had hypergammaglobulinemia and positive Coombs tests. Development of lymphadenopathy, splenomegaly, and autoantibodies during follow-up in children with cITP and AIHA should alert the physician to a possible diagnosis of ALPS.

Lymphoma is usually diagnosed in patients with ALPS at advanced ages. Lymphoma at a median age of 17 years in both adults and children with ALPS was reported in one study [8]. However, the median age of lymphoma patients was found as 12 years in our study. Most reported patients with ALPS have Hodgkin lymphoma, and EBV is positive in these cases [15,16]. Similarly, our patients were mostly diagnosed with Hodgkin lymphoma, but investigations of these patients revealed only two cases with EBV. In addition, children with newly diagnosed lymphoma with the presence of anemia and thrombocytopenia were approximately 3- to 4-fold more likely to develop ALPS. Our study indicated that the presence of anemia and thrombocytopenia in patients with lymphoma at diagnosis may be useful for ALPS screening.

Splenectomy and rituximab are not recommended in ALPS because of sepsis and recurrence risk in most cases [1,2,3,21,22,23,24]. We canceled the scheduled splenectomies for five patients with massive splenomegaly. Furthermore, some patients with cITP and AIHA might be resistant to standard treatment, as in previous reports [25,26]. Partial response to rituximab was observed in cases of AIHA. We believe that treatment response could help the physician reach a possible diagnosis of ALPS in children with cITP, AIHA, and nonmalignant organomegaly. The major limitations of the present study were that the other ALPS-related genes [27,28,29,30] were not studied due to lack of resources and all lymphoma cases/adult cases were not included.

Our data indicate that investigation of ALPS is warranted in children with lymphoma presenting with cytopenia, in cases of chronic nonmalignant organomegaly with immune cytopenia, and probably in patients with cITP and AIHA developing organomegaly during follow-up.

## Figures and Tables

**Table 1:**
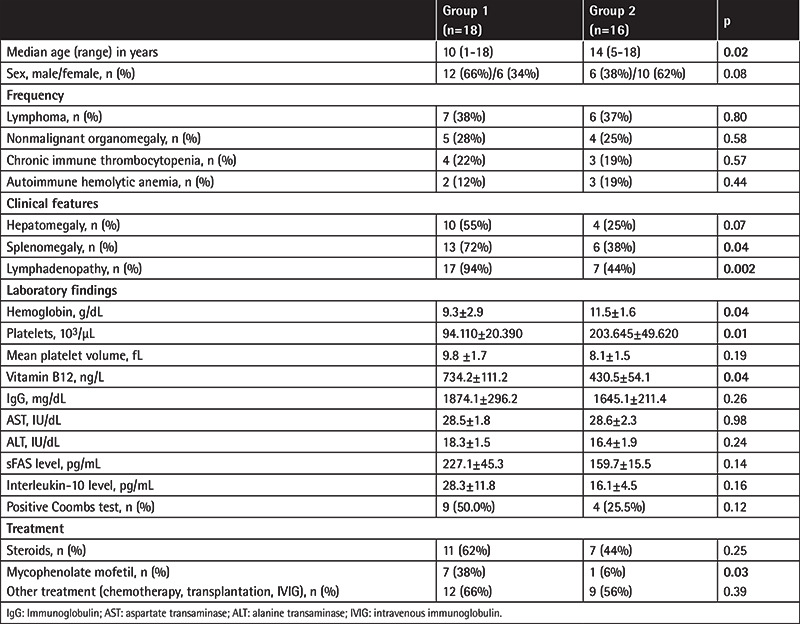
Demographic characteristics, frequency, clinical features, and laboratory parameters for the patient groups.

**Table 2:**
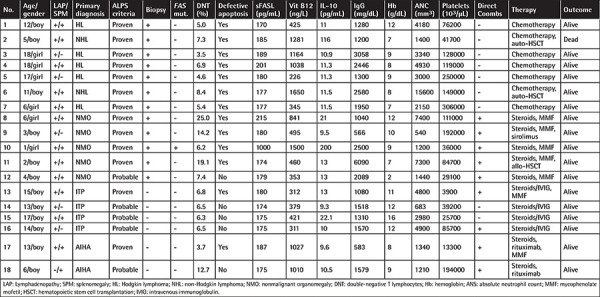
The clinical and laboratory findings and outcomes in proven and probable patients with autoimmune lymphoproliferative syndrome.
